# Lack of Viral Load Within Chronic Lymphoproliferative Disorder of Natural Killer Cells: What Is Outside the Leukemic Clone?

**DOI:** 10.3389/fonc.2020.613570

**Published:** 2021-01-18

**Authors:** Edoardo Giussani, Andrea Binatti, Giulia Calabretto, Vanessa Rebecca Gasparini, Antonella Teramo, Cristina Vicenzetto, Gregorio Barilà, Monica Facco, Alessandro Coppe, Gianpietro Semenzato, Stefania Bortoluzzi, Renato Zambello

**Affiliations:** ^1^ Department of Molecular Medicine, University of Padova, Padova, Italy; ^2^ Department of Medicine, Hematology and Clinical Immunology Branch, University of Padova, Padova, Italy; ^3^ Veneto Institute of Molecular Medicine (VIMM), Padova, Italy; ^4^ Department of Maternal and Child Health, University of Padova, Padova, Italy; ^5^ Department of Biology, University of Padova, Padova, Italy; ^6^ CRIBI Biotechnology Centre, University of Padova, Padova, Italy

**Keywords:** natural killer cells, chronic lymphoproliferative disorder of natural killer (NK) cells, retroviral infection, killer immunoglobulin-like receptors, pneumocytes, hepatocytes

## Abstract

Large granular lymphocyte leukemias (LGLL) are sustained by proliferating cytotoxic T cells or NK cells, as happens in Chronic Lymphoproliferative Disorder of Natural Killer cells (CLPD-NK), whose etiology is only partly understood. Different hypotheses have been proposed on the original events triggering NK cell hyperactivation and transformation, including a role of viral agents. In this perspective, we revise the lines of evidence that suggested a pathogenetic role in LGLL of the exposure to retroviruses and that identified Epstein Barr Virus (EBV) in other NK cell leukemias and lymphomas and focus on the contrasting data about the importance of viral agents in CLPD-NK. EBV was detected in aggressive NK leukemias but not in the indolent CLPD-NK, where seroreactivity against HTLV-1 retrovirus envelope BA21 protein antigens has been reported in patients, although lacking clear evidence of HTLV infection. We next present original results of whole exome sequencing data analysis that failed to identify viral sequences in CLPD-NK. We recently demonstrated that proliferating NK cells of patients harbor several somatic lesions likely contributing to sustain NK cell proliferation. Thus, we explore whether “neoantigens” similar to the BA21 antigen could be generated by aberrancies present in the leukemic clone. In light of the literature and new data, we evaluated the intriguing hypothesis that NK cell activation can be caused by retroviral agents located outside the hematopoietic compartment and on the possible mechanisms involved with the prospects of immunotherapy-based approaches to limit the growth of NK cells in CLPD-NK disease.

## Introduction

Chronic Lymphoproliferative Disorder of Natural Killer cells (CLPD-NK) belongs to the group of large granular lymphocyte leukemias (LGLL), sustained by cytotoxic T cells (T-LGL) or NK cells. CLPD-NK is a rare disease characterized by the persistence of NK cell lymphocytosis, typically presenting a restricted pattern of activating killer immunoglobulin like receptors (KIR) (1). Several lines of evidence point to a putative role of viral agents in the pathogenetic mechanisms sustaining NK cell proliferation (2). Implication of exogenous factors in LGLL has been recently suggested also by Nyland et al. (3), demonstrating the exposure to a retrovirus in both patients and clinically normal contacts sharing the same environment.

A pathogenetic role for Epstein Barr Virus (EBV), identified several years ago (4), was recently supported by data (5, 6) regarding the severe forms of NK cell proliferation, *i.e.* Aggressive NK Cell Leukemia (ANKL) and Nasal NK/T-Cell Lymphoma (NKTCL). Anyhow, EBV DNA was seldom detected by PCR in the indolent CLPD-NK (7, 8). Moreover, seroreactivity against HTLV-1 envelope (env) protein antigens has been reported in CLPD-NK, although patients were not demonstrated to be infected with a prototypical HTLV retrovirus (9). At present, even though an implication of viruses in the pathogenesis of CLPD-NK is highly suspected, conclusive data on a direct role for exogenous viral agents as transforming factors within NK cells in CLPD-NK patients are lacking.

## Lack of Viral Load in Chronic Lymphoproliferative Disorder of Natural Killer Cell Patients

Several studies showed that Whole Exome Sequencing (WES) data of cancer cells can be useful to identify pathogens, particularly viruses, in cancer clones and also to define possible sites of integration in the host genome or their presence in episomal forms (10, 11). In both ANKL and NKTCL, a larger EBV load in tumor clones than control cells of the same patient has been detected by WES (11). In addition, frequent focal EBV genome deletions and integrated EBV fragments in the host genome have been found in NKTCL (12).

**Figure 1:**
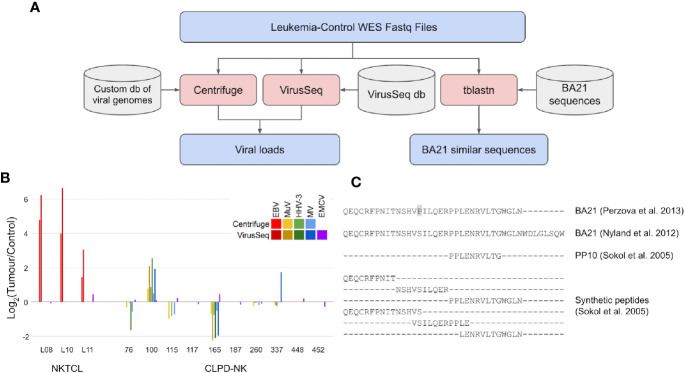
Search for viral load and neoantigens similar to BA21 in WES data of CLPD-NK patients. **(A)** Analyses workflow for viral load and BA21 neoantigens. Files are indicated in blue, bioinformatic tools in red and databases in grey. **(B)** Load of viral sequences detected by Centrifuge and VirusSeq in tumor and control WES data of patients with NKTCL (11) and with CLPD-NK (13); for each patient, the viral load is expressed as Log2 of CPM ratio in tumor *versus* control samples; EBV, Epstein Barr virus, MuV, mumps virus, HHV-3, human alphaherpesvirus 3, MV, measles virus, EMCV, encephalomyocarditis virus; **(C)** Alignment of BA21 antigen sequences collected from literature (14, 15), of the shortest antigenic peptide PP10 and of other synthetic peptides covering BA21 sequence (16) that were compared with WES data to identify neoantigens.

We performed a very thorough research for viral DNA and neoantigens from WES data of 10 patients with CLPD-NK recruited at the Hematology Unit of Padua University Hospital and recently investigated for somatic mutations (13) (**Figure 1A**). The study and blood sample collection were approved by the Ethic Committee for Clinical Trials of Padua. CLPD-NK diagnosis followed WHO criteria, and all patient data can be found in the original study. Briefly, these patients were characterized by clonal expansion of NK-cells ≥0.5 × 10^9^/L lasting more than 6 months, typical immunophenotype and absence of STAT3/STAT5b mutations. High depth WES data (51 M reads in average) were obtained by highly purified tumoral NK cells and control autologous granulocytes from each patient. All tumoral and control samples were analyzed using all the sequence reads from FASTQ files that passed quality check. In this way, both patients’ sequences aligning and not aligning to the reference human genome were considered in exhaustive analyses contemplating that in a patient’s genome several factors, including genetic aberrancies and virus integration, could generate sequences different from the reference genome.

To intercept viral DNA from NGS data, we used two bioinformatic methods for pathogen discovery, Centrifuge (17) and VirusSeq (18), both based on large databases of viral genomes but implementing different strategies to identify and quantify viral DNA as counts per million reads (CPMs). Centrifuge analysis was performed using a custom version of the provided database, with only human and viral sequences, while VirusSeq was run with its default database of virus genomes. The DNA of a specific virus was considered detected if the identified viral sequences were quantified with Centrifuge CPM >1 or VirusSeq CPM >5, which are comparable considering the different reference viral sequence sets used and alignment methods implemented in the two software tools. Centrifuge and VirusSeq were firstly tested by re-analysis of WES data in three NKTCL patients from Dufva et al. (11), obtaining data consistent with the two methods (**Figure 1B**) and confirming previous findings of an EBV load in tumor clones (CPM = 14, in average) higher than in control samples (average log_2_ tumor/control CPM = 3.5, comparing tumor and control samples of the same patient). Differently, analysis of WES data of the 10 CLPD-NK patients with the same methods did not identify EBV or other viruses previously associated with human cancer (**Figure 1B**). Four human viruses were detected: human alpha-herpesvirus 3 (Varicella Zoster), mumps virus, measles virus, and encephalomyocarditis virus, all at low absolute loads (about ten times lower than EBV loads detected in NKTCL samples). The first three viruses were found in six out of 10 CLPD-NK patients; encephalomyocarditis virus was detected in all samples. Importantly, the load of all the detected viruses was similar in tumor and control samples. In summary, our analyses of WES data with two methods consistently did not detect sequences of cancer-associated viruses in CLPD-NK patients and did not report an increased load in leukemic clones for the few detected viruses. With the limit of low number of patients analyzed, these data, even if not completely ruling out the role of previous infections in CLPD-NK, did not support the hypothesis of a viral load specifically in the tumor clone.

## Genome Analysis Did Not Detect The Presence of Ba21-Like Neoantigens in Cancer Clones of Chronic Lymphoproliferative Disorder of Natural Killer Cell Patients

We considered that a peculiar feature reported for the majority of CLPD-NK cases is represented by seroreactivity against BA21, a peptide derived from HTLV-I envelope protein p21 (9). Previous analysis of synthetic peptides representing BA21 fragments revealed that the minimal epitope responsible for seroreactivity of patients with T-LGLL is a decapeptide (PP10, p21 env 417–426) lacking significant similarity with any known human protein (16).

However, CLPD-NK patients were seldom exposed to HTLV-I before the disease, lacking prototypical HTLV-I and HTLV-II infection (19). New evidence of somatic mutations (single nucleotide polymorphisms, insertions and deletions) harbored by tumor clones of CLPD-NK patients (13) has awakened the open question of whether an altered proteome in CLPD-NK patients could contribute to the disease also by endowing cancer cells with aberrant antigenic peptides (cancer neoantigens) inciting or sustaining NK proliferation. WES data of CLPD-NK patients were thus scrutinized for evidence of endogenous “neoantigens” similar to viral antigens for which patient seroreactivity was previously described. All the BA21 sequences available in literature were compared, using tblastn tool (20), against a nucleotide blast database made from WES reads of CLPD-NK tumor and control samples (**Figure 1A**). In particular, in this comparison we considered the complete BA21 epitope (14, 15), PP10, the shortest antigenic peptide derived from BA21, and six other overlapping peptides covering the entire BA21 epitope (16) (**Figure 1C**). The genomic sequences of CLPD-NK patients with the highest similarity with BA21 peptides aligned with many mismatches and gaps. At most five contiguous amino acid matches were found between patient-derived WES sequence reads and BA21 peptide sequences. Sequences very similar to the antigenic PP10 or part of it were not found. Our analysis of CLPD-NK patient exomes did not identify any sequences capable of generating a neoantigen similar to BA21 epitope.

## Chronically Sustained Natural Killer Proliferation: A Role of the Interplay Between Natural Killer Cells and Virus-Infected Cells Outside the Hematopoietic Compartment?

Collectively, our new data do not support an intrinsic role of a viral infection of NK cells or of intrinsic aberrant neoantigens in the leukemic clone in maintaining the NK cell proliferation in CLPD-NK. Interestingly, LGLs (both T and NK) have been recently scrutinized for inserted endogenous retroviruses (namely HERV-Ks) without detecting any new retroviral integration sites in the clonally expanded cells, but finding a higher burden of polymorphic HERV-K provirus in LGLL patient genomes (21). Although no prototypical seroreactivity to HERV-K Gag or Pol antigens could be found in LGLL patients, possibly an antibody response might be directed towards antigens expressed by cells different from proliferating NK cells, hosting and exposing epitopes of an aberrantly expressed HERV-K. A viral infection outside proliferating NK cells might play a role as a starting event for inciting and possibly maintaining NK cell proliferation. Although a specific cell type has not been caught red-handed yet, cells from different sites have been previously demonstrated to be targets of retroviral infections. Pneumocytes (22) and also hepatocytes are a well-recognized target of NK cells in LGLL patients (23, 24). Lamy and colleagues for the first time provided evidence of a selective damage of pneumocytes and hepatocytes from pathological LGL in patients with LGL leukemia. In this condition, reminiscent of graft-*versus*-host disease, a direct attack mediated by LGL takes place and ultimately leads to cell apoptosis. Clinically, an extreme condition has been described, in which Pulmonary Hypertension and LGL leukemia are associated (25), despite being both very rare. Other case reports indicated lung or liver as main targets of LGL (26, 27). Beyond this extreme condition, a subtle initial damage mediated by LGL to hepatocytes and pneumocytes, in which NK cells recognize retroviral antigens, can be in place but go unnoticed in patients. In addition, considering the well-established ability of the infectious retroviruses to activate and recombine with HERVs (28, 29), it can be postulated that proliferating NK cells from CLPD-NK patients might be stimulated by antigens from a chimeric, replication-incompetent retrovirus located outside NK cells. The search of antigens processed by dendritic cells within the bone marrow is also encouraged by the peculiar interaction between LGL and dendritic cells (30). Taken together, we propose that the investigation outside the hematopoietic compartment for the presence of BA21 antigen sequence might represent an interesting issue to pursue in the future.

## The Possible Contribution of Activating Killer Immunoglobulin Like Receptors to Natural Killer Cell Proliferation

**Figure 2:**
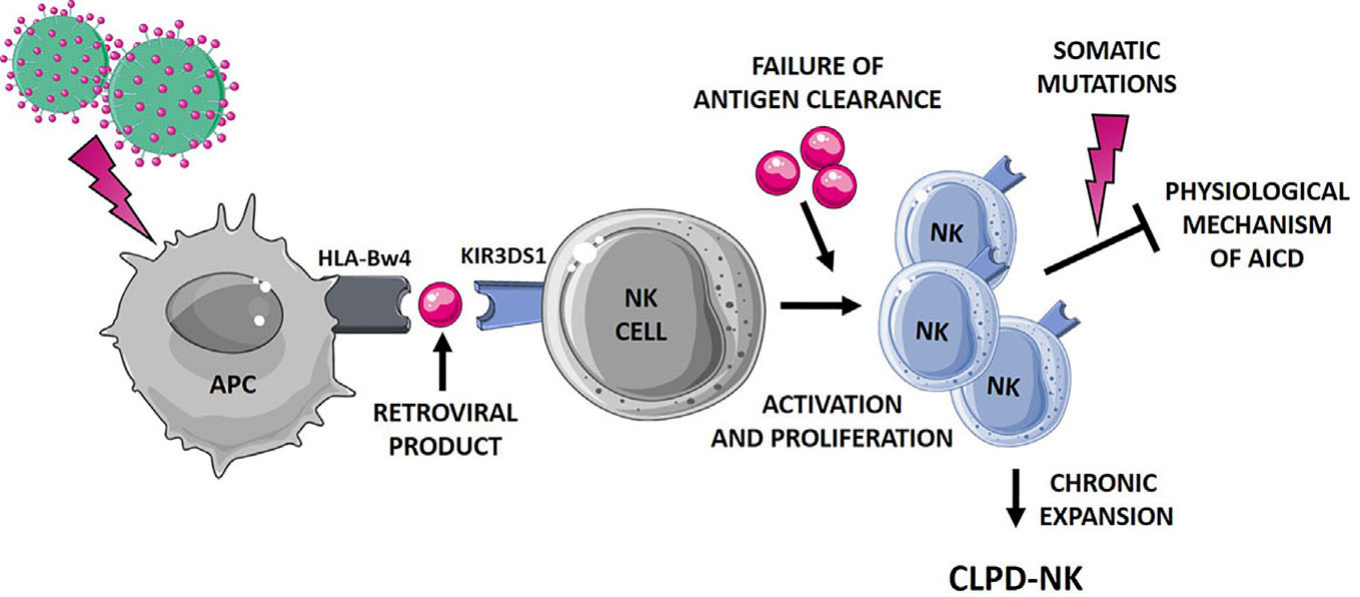
Hypothetical mechanisms favoring NK cell proliferation leading to CLPD-NK. The first step of NK cell activation and proliferation can be related to chronic retroviral stimulation. NK cells, which mostly express the activating KIR3DS1, might interplay with antigen presenting cells (APC). According to literature (31), a small peptide, as a retroviral product, might be involved in this interaction. The failure of antigen clearance might lead to the maintenance of an aberrantly activated NK cell clone, resistant to the physiological mechanism of activation induced cell death (AICD). Additional events, such as the occurrence of somatic mutations (13), can further promote NK cell survival, leading to the establishment of a chronic expansion of clonal NK cells. APC, antigen presenting cells; AICD, activation induced cell death. The figure was created using Smart Servier Medical Art.

Differently from T-LGLL, CLPD-NK is sustained by NK cells that lack specific rearranged receptors leading to a fine antigen recognition. Thus, evaluation of how NK cells do recognize specific antigens is needed to shed some light on the mechanisms underlying aberrant NK activation in CLPD-NK (**Figure 2**).

We showed that in the majority of patients with CLPD-NK, dominant KIRs trigger an activating signal, suggesting that activating KIRs are the targets of the inciting event leading to LGL proliferation (1). NK cell education to tolerate the self prevents activation against normal tissues. This process, termed “licensing”, acts through an MHC-dependent mechanism requiring interaction between inhibitory KIR and cognate MHC class I ligands. Recent data indicate that also activating KIRs play a role, previously underestimated, in NK licensing. Activating KIRs are also involved in the control of some viral infections *in vivo*. We demonstrated that *KIR3DS1* (usually present in 60% of normal Caucasian individuals) is expressed in the majority of CLPD-NK patients, whereas *KIR3DL1*, that is usually present and expressed in the majority (90%) of normal individuals, is switched off in CLPD-NK patients through the methylation of its promoter (32). KIR3DS1 expression is associated with the outcome of various diseases, including viral infections, malignancies and autoimmune diseases (33). KIR3DS1 recognizes peptides modulated by HLA-Bw4 alleles playing a crucial role in NK cell education (34), and the highest cytotoxic potential was detected when Bw4I80 is lacking. Indeed, in this regard, we previously showed that HLA-I/KIR mismatch is often present in NK cell proliferation in CLPD-NK (2, 35). HLA-F in its open conformer status is a KIR3DS1 ligand (36, 37) expressed on the surface of some EBV-transformed B (38) and up-regulated in HCV infected liver cells that are subsequently sensed by KIR3DS1 (39). Since we previously showed that HLA-I/KIR mismatch is a typical feature in CLPD-NK (2, 35), HLA-F should be evaluated as a possible target of KIR3DS1, even if lacking specific HLABw4 alleles.

Further, it has been suggested that the binding HLA-KIR might be dependent or modulated by a third component, namely a small peptide endowed in HLA groove (31). Interestingly, such a mechanism might not be restricted to KIR3DS1, since it has been demonstrated for other activating KIRs (40) and for inhibitory KIRs as well (41). All these mechanisms might contribute to activate and expand NK cells, but likely do not lead to the clone immortalization and to the resistance to the Activation Induced Cell Death. An additional step might concur to the persistence of the NK clone, as the inability of antigen clearance and/or to the occurrence of new events, including somatic mutations. As a matter of fact, despite the relatively “benign” and indolent nature of the CLPD-NK disease, a high number of deleterious somatic lesions, impacting important cellular functions, were recently detected in this condition (13). The leukemic clone of CLPD-NK patients carried a heavy burden of lesions involving genes related to cancer proliferation, survival and migration, thus sustaining the hypothesized mechanism. **Figure 2** summarizes the hypothetical mechanisms favoring NK cell proliferation leading to CLPD-NK.

## Conclusions

In this perspective, taking advantage from our original data confirming with a sensitive technique the lack of viral load in NK cells from CLPD-NK patients, we suggest that retroviral infection possibly targeting cells outside the hematopoietic compartment might lead to an immune response causing on one hand, the production of anti BA21 env related antibodies and on the other hand, an immune attack mediated by LGL on these target cells. More specifically, our WES data analysis, looking for the presence of exogenous and endogenous viral material in proliferating NK cells of CLPD-NK patients, did not support a direct role of an integrated or episomal viral agent in NK cells. Neither, somatic lesions of CLPD-NK seem to be able to generate neoantigens similar to the BA21 peptides for which seroreactivity was described in patients. After careful revision of literature data, pneumocytes or hepatocytes can be suggested among possible viral targets, inciting NK cells attack. The involvement into this process of activating KIRs, particularly KIR3DS1, is supported by available evidence, indicating as well that small antigenic peptides can be presented by these receptors. This hypothesis must be confirmed in order to explore the possibility to limit the growth of NK cells by immunotherapy and develop a new curative approach for CLPD-NK, a disease for which efficient specific therapy still remains an unmet need.

## Data Availability Statement

The data analyzed in this study is subject to the following licenses/restrictions: WES data are available upon request. Requests to access these datasets should be directed to RZ, r.zambello@unipd.it.

## Ethics Statement

The studies involving human participants were reviewed and approved by the Ethic Committee for Clinical Experimentation of Padua (Comitato etico per la sperimentazione clinica della provincia di Padova)—Padua Hospital. The patients/participants provided their written informed consent to participate in this study.

## Author Contributions

EG, AB, GC, GS, RZ, and SB conceived the study. VRG, AT, CV, and GC prepared the samples for the WES analysis. EG, AB, AC, and SB contributed bioinformatics methods and made the data analysis. GB, MF, GS, and RZ provided patient samples. EG, AB, SB, and RZ wrote the paper. EG, AB, SB, and GC made the figures. GS provided funding and critically reviewed the manuscript. All authors contributed to the article and approved the submitted version.

## Funding

This study was supported by the Associazione Italiana per la Ricerca sul Cancro, Milano, Italy (IG #20216 to GS and IG #20052 to SB), and Italian Ministry of Education, Universities and Research (PRIN #2017PPS2X4_003 to SB).

## Conflict of Interest

The authors declare that the research was conducted in the absence of any commercial or financial relationships that could be construed as a potential conflict of interest.
